# Estimating the returns to United Kingdom publicly funded musculoskeletal disease research in terms of net value of improved health outcomes

**DOI:** 10.1186/s12961-017-0276-7

**Published:** 2018-01-10

**Authors:** Matthew Glover, Erin Montague, Alexandra Pollitt, Susan Guthrie, Stephen Hanney, Martin Buxton, Jonathan Grant

**Affiliations:** 1 Health Economics Research Group, Brunel University London, Uxbridge, United Kingdom; 2 Policy Institute at King’s, King’s College London, Virginia Woolf Building, 22 Kingsway, London, WC2B 6LE United Kingdom; 3 RAND Europe, Cambridge, United Kingdom

**Keywords:** Medical research investment, QALYs, Musculoskeletal disease, Medical research charities, Value of health, Rate of return, Elapsed time, Research payback

## Abstract

**Background:**

Building on an approach applied to cardiovascular and cancer research, we estimated the economic returns from United Kingdom public- and charitable-funded musculoskeletal disease (MSD) research that arise from the net value of the improved health outcomes in the United Kingdom.

**Methods:**

To calculate the economic returns from MSD-related research in the United Kingdom, we estimated (1) the public and charitable expenditure on MSD-related research in the United Kingdom between 1970 and 2013; (2) the net monetary benefit (NMB), derived from the health benefit in quality adjusted life years (QALYs) valued in monetary terms (using a base-case value of a QALY of £25,000) minus the cost of delivering that benefit, for a prioritised list of interventions from 1994 to 2013; (3) the proportion of NMB attributable to United Kingdom research; and (4) the elapsed time between research funding and health gain. The data collected from these four key elements were used to estimate the internal rate of return (IRR) from MSD-related research investments on health benefits. We analysed the uncertainties in the IRR estimate using a one-way sensitivity analysis.

**Results:**

Expressed in 2013 prices, total expenditure on MSD-related research from 1970 to 2013 was £3.5 billion, and for the period used to estimate the rate of return, 1978-1997, was £1.4 billion. Over the period 1994–2013 the key interventions analysed produced 871,000 QALYs with a NMB of £16 billion, allowing for the net NHS costs resulting from them and valuing a QALY at £25,000. The proportion of benefit attributable to United Kingdom research was 30% and the elapsed time between funding and impact of MSD treatments was 16 years. Our best estimate of the IRR from MSD-related research was 7%, which is similar to the 9% for CVD and 10% for cancer research.

**Conclusions:**

Our estimate of the IRR from the net health gain to public and charitable funding of MSD-related research in the United Kingdom is substantial, and justifies the research investments made between 1978 and 1997. We also demonstrated the applicability of the approach previously used in assessing the returns from cardiovascular and cancer research. Inevitably, with a study of this kind, there are a number of important assumptions and caveats that we highlight, and these can inform future research.

**Electronic supplementary material:**

The online version of this article (doi:10.1186/s12961-017-0276-7) contains supplementary material, which is available to authorized users.

## Background

Total global investment in biomedical and health research was estimated at US$240 billion in 2009 [1], equivalent to approximately US$270 billion in 2016. These investments are intended to improve health for patients and the public. But do they? And if so, what are their returns?

In recent years, researchers and research funders have aimed to better understand the range of impacts arising from public and charitable funding for medical research, including the resulting economic benefits. Such information provides accountability to taxpayers and charity donors, and increases our understanding of how research effectively translates to health gains. In this paper, we examine the economic returns from musculoskeletal disease (MSD) research. This is the third in a series of studies looking at the returns from cardiovascular (CVD) research [2] and cancer research [3], as well as the broader economic impacts or spillover effects of research funding [4].

As reviewed by Buxton et al. [5], and updated by Glover et al. [3] and Raftery et al. [6], the literature that assesses the value of the benefits of medical research forms a relatively limited field in terms of methodology and quality. There are two broad approaches. Firstly, a ‘top down’ approach where overall health gains in a disease area are related to research investments, but this requires an estimate of how much of the total health gain can be attributed to medical research investments. For example, Funding First [7] argued, in a report entitled ‘Exceptional Returns’, that the steep decline in CVD deaths in the United States between 1970 and 1990 had an economic value of US$1.5 trillion annually, and deduced that one-third of this (US$500 billion a year) could be attributed to medical research that led to new procedures and drugs. The approach was replicated in a series of studies by Access Economics [8, 9] and Deloitte Access Economics [10] estimating the return on Australian biomedical research on the basis of overall improvements in Australian lifespan. The base-case assumption in these studies was that research was responsible for 50% of the improvements in healthy lifespan, although it is worth noting that the authors acknowledged there was no evidence to support this assumed rate of attribution.

The challenge of top-down attribution can be addressed by examining in a ‘bottom-up’ manner the impacts of specific projects or programmes of research by tracing forwards from the research to the benefits that arise. This is the approach developed by HERG [2] and Glover et al. [3], and adopted in this study. Here, we estimate the net monetary benefits (NMB), defined as the health benefit valued in monetary terms minus the cost of delivering that health benefit, for a set of key interventions to reduce MSD that arose from the United Kingdom application of relevant United Kingdom research. This ‘bottom-up’ approach led to an impressive but less ‘exceptional’ internal rate of return (IRR) of 9% and 10% for CVD and cancer research, respectively [2, 3].

However, in both these studies the reduction of smoking over the period analysed had a major impact on the estimated rate of return. For example, the return on cancer research investment declined to 2.4% in a sensitivity analysis that excluded the effect of smoking cessation, and attribution of the reduction in smoking to medical research alone is contestable. Glover et al. [3] therefore concluded it would be valuable to undertake an investigation in another clinical area, such as MSD, in which smoking only marginally affects outcome to see whether similar rates of return are found [3] (smoking has a comparatively small effect on the musculoskeletal system, including a reduction in bone mineral content and deleterious effects on osteoporosis, fractures and other MSD [11, 12]). Another prima facie, methodological reason why MSD research is an interesting case to examine is that it largely relates to chronic conditions, where health gains occur through improvements in morbidity, rather than mortality as was the case for CVD and cancer.

### The MSD burden of disease and research

How much biomedical and health research funding is invested in different disease areas is determined by a number of factors, including burden of disease, scientific tractability, donor appeal and previous investment [13]. The United Kingdom Clinical Research Collaboration [14] report a relatively weak correlation between research investment and burden of disease (using disability adjusted life years (DALYs)), using the health categories in the Health Research Classification System (HRCS).^1^ Whilst ‘cancer’ has the highest proportion of spend and highest DALY rate (ca. 20%), the combined health research categories ‘cardiovascular’, ‘blood’ and ‘stroke’, have approximately 16% of burden, but only 9% of the spend. ‘Musculoskeletal’ has an even greater skew, with approximately 9% of the burden but only 3% of spend.

MSD has relatively low rates of mortality, although evidence indicates incidences of deaths in which MSD conditions were the underlying cause of death are under-reported [15, 16]; however, it has a relatively high prevalence of disability and morbidity. Many musculoskeletal conditions are recurrent and lifelong disorders which can often cause long-term pain, physical disability, loss of independence, reduced social interaction and a decline in quality of life [17]. Arthritis conditions, for example, are the biggest cause of pain and disability in the United Kingdom [16].

While most MSD conditions do not require hospital admission, MSDs are a frequent cause of consultation with general practitioners (GP). For example, 15–20% of all GP consultations involve a patient with MSD conditions [17]. Further, Woolf et al. [18] found, in a cross-national comparison, that MSD conditions are one of the leading causes of both long-term absences from work and disability pension claims.

MSD conditions affecting joints, bones, muscles and soft tissues can affect any age group, but the prevalence of the disease increases drastically for older people. The age group most commonly affected (50+ years old) tends to fall predominately outside of the active labour force. Further, conditions which fall under MSD affect approximately 10 million people in the United Kingdom, accounting for £5 billion of the NHS programme budget spend in England alone [16].

Therefore, MSD has a very different funding and disease profile to that of the two previous studies on CVD [2] and cancer [3].

### Defining the scope of MSD

For this study, we needed a clear and internationally defensible definition of ‘musculoskeletal’ disease. Following consultation with a number of experts (see acknowledgements) we used Chapter XIII of the 10th revision of the International Statistical Classification of Diseases and Related Health Problems (ICD), known colloquially as ICD 10 Chapter XIII [19]. One advantage of using Chapter XIII of ICD 10 is that it is also the basis of the musculoskeletal category in the HRCS, meaning that, in many cases, research investment and health outcomes are defined using the same criteria.

As discussed in more detail below, we focussed on five condition groups/areas (with the number indicating the ICD sub-classification):

- Inflammatory arthritis (M00–M14): particularly rheumatoid arthritis (RA), juvenile idiopathic arthritis (JIA), ankylosing spondylitis, psoriatic arthritis and gout
- Osteoarthritis (M15–M19)
- Connective tissue disorders (M30–M36): particularly systemic lupus erythematosus (SLE) and dermatomyositis
- Back pain and dorsopathies (M40–54)
- Osteoporosis (M80–82)

## Methods

### Overall approach

**Fig. 1:**
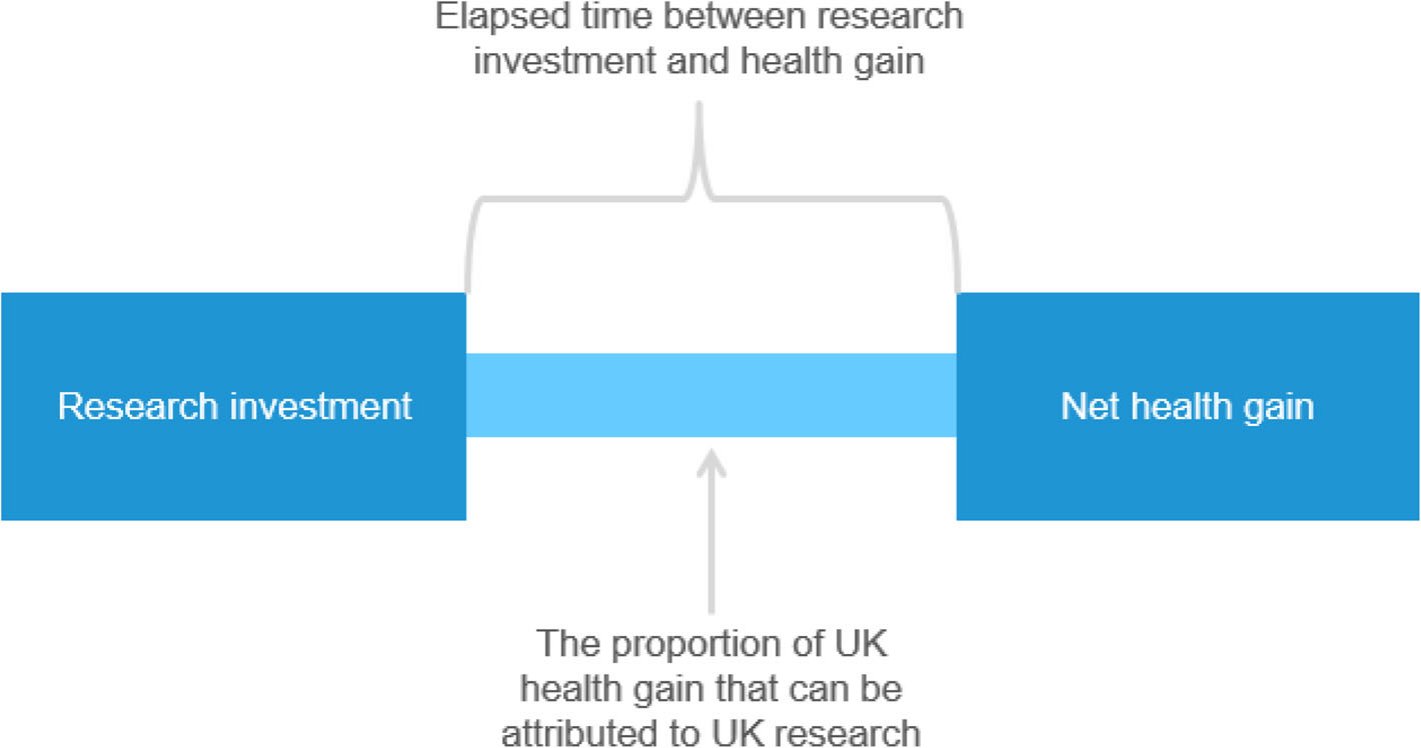
Overall study approach

The overall conceptual approach is summarised in Fig. 1 and requires four key data elements to estimate the IRR arising from MSD research, namely (1) a time series of public and charitable funding of MSD-related research; (2) a time series of NMB of MSD health gains, derived from the monetised health benefits and healthcare costs from the actual use of selected interventions; (3) an estimate of the elapsed time between the investment (research funding) and return (health gain) associated with those interventions; and (4) an estimate of the amount of health gain that should be attributed to United Kingdom public and charitable research investment in MSD-related research.

With these four data inputs, the IRR on the public and charitable investment in MSD research and development (R&D) can be calculated (it should be noted that the costs of private sector R&D investments are accounted for in our analysis as elements within the cost of delivering healthcare, which are netted off in the NMB). The costs to the health service of medical interventions produced by the private sector are assumed to include the return to the private sector on its R&D investments.

### Estimating public and charitable funding of MSD-related research

We developed a time series of public and charitable funding of MSD-related research between 1970 and 2013 for the five largest research funders and a group of other research charities. Each involved a different approach.

### Medical Research Council (MRC)

The MRC had previously provided digital copies of its annual reports dating back to 1911. Between 1976 and 1992, the MRC used a consistent disease classification system for its research grants, including the category ‘Muscle, Bone and Joints’, which we used for this study. Data from 1976 to 1992 was extracted from heading 2 of the annual reports, which classifies projects according to relevance – that is, the total spend on each project is placed against any (and all) relevant categories (i.e. it can be double counted). In comparison, this classification method is more ‘inclusive’ than the alternative first heading in the MRC annual reports, which uses a classification according to the primary purpose, that is, the total spend equates to the total MRC spend for the year. As with the previous studies we use this broader definition of expenditure as it is likely to overstate funding and thus err on the side of being conservative when calculating the rate of return. In addition, the MRC gave access to annual reports detailing programme expenditure on conditional area ‘Musculoskeletal’ research from 2009 to present day, based on the HRCS definition.

Information was not available between the periods of 1970 to 1975 and 1993 to 2008, in which case we calculated missing data by inter- and extrapolating the missing data using various growth functions in Excel.

### Wellcome Trust (WT)

The WT produced a detailed list of the grants awarded from 1970 to present day, and a summary of the total commitment annually. The WT grant management system identified awards with a 25% or higher proportion classified as ‘musculoskeletal’, based on the HRCS definition.

Once data was compiled in Excel it was analysed for patterns and irregularities. For example, in 2008, there was an increase of approximately £28 million in total commitment as a result of grants awarded for three long-term research programmes, all in biomedical engineering. Upon request, WT provided further grant descriptions on the proportion of grants which had been classified as less than 50% ‘Musculoskeletal’ to determine whether the grant was within scope of the disease area as defined by ICD 10, and we reduced the total commitment to reflect the proportion of the grant falling under this classification.

To adjust the total commitment data into total expenditure, we assumed an average of 3 years for each grant and allocated commitment over this time period and re-calculated expenditure on a per year basis.

### Arthritis Research United Kingdom (ARUK)

We assumed all ARUK research expenditure related to MSD. ARUK provided us with detailed data on the total commitment as recorded in their income and expenditure statements, up to and including 2008, and total expenditure from 2009 onwards.

Once data was collected and collated into an Excel spreadsheet, it was analysed for irregularities. The data showed a significant decrease in 2002, which mainly resulted from a change in research strategy, and therefore a pause in the funding of new grants.

As with the Wellcome Trust we adjusted commitment data (between 1970 and 2008) by assuming an average grant length of 3 years and allocating expenditure per year on that basis.

### MSD research activity index

The Department of Health (DH) and Funding Councils (FCs) did not record information on research funding by disease area. As with the previous studies, we were able to generate a total expenditure for both, as described below, and multiplied this by an ‘activity index’ to estimate the amount of research expenditure on MSD annually.

The activity index was estimated by looking at the total expenditure on MSD research by the MRC and the WT and by comparing it with bibliometric data that was commissioned to inform other elements of the study. We also compared it to other sources, including a historical analysis of NHS research, which suggests that 4.5% of research outputs were related to MSD research [20], and a more recent analysis using the HRCS, which suggests that 2.8% of research spend by the top 12 public funders in the United Kingdom is on MSD research [14]. Overall, we assumed that 3% of all biomedical and health research activity is related to MSD research, ranging between 2% and 4% for sensitivity analysis.

### Department of Health (DH)

The DH did not have information available on total MSD research spend, nor did they have data on the total spend from one source. Additionally, we were interested in estimating the total research spend by the DH as well as the National Health Service (NHS), collectively. Therefore, as with the previous studies, we collected data from three sources, namely data for 1973 was entered by hand from Maddock, 1975 [21]; data for 1981 to 1984 was entered by hand from the Annual Review of Government Funded R&D, 1984; and data on the DH (excluding NHS) from 1986 onwards data were collected from SET statistical table 3.1 for Department of Health and Social Security and the DH. NHS funding was included from 1995 onwards and subsequent NIHR data from its founding in 2006.

Data was not available for either NHS or the DH for 1970–1972 or 1974–1980. Further, data for the NHS could not be extracted prior to 1995. Therefore, we estimated the expected funding in Excel for both NHS and DH separately to provide a time series for each, and added the two estimates for the total expenditure. We then multiplied our total DH/NHS/NIHR funding series by the activity index (as described above) to generate an estimate of total DH funding in MSD research.

### Funding Councils (FCs)

Similar to the DH, the FCs did not differentiate research spend by field area, and therefore data on total research spend was collected and/or estimated and multiplied by the research activity index (see above). Data was collected from 1989 onwards from three sources, namely for 1989–1992 from the Research Grant figures for Great Britain, provided by the HEFCE^2^; funding from 1993 to 2008 was extracted from the HEFCE mainstream quality-related research grant allocations for biomedical subjects in the years 1993–1994 to 2008–2009^3^; and funding from 2009 to 2012 was provided through the HEFCE mainstream quality-related research grant allocations for biomedical subjects in the years 2009–2010 to 2012–2013.^4^

Data could not be extracted from 1970 to 1988; therefore, we took a similar approach to that used for DH funding. We projected the best linear fit of data for the period 1988 to 2012, then determined the expected growth from the same time series in order to estimate for missing annual data.

### Other medical research charities

In addition to WT and ARUK, we were aware of other medical research charities that supported MSD research. We therefore approached the Association of Medical Research Charities (AMRC), which helped us identify and select that ‘other group’. Using the HRCS report for 2014 [14], AMRC identified 21 members who funded MSD research. Two of these charities were out of scope (because they were funding research outside the United Kingdom), leaving 19 AMRC members with a total spend of approximately £18 m per year on MSD research. When WT and ARUK were excluded, the remaining 17 charities spent approximately £2 m annually, with the top nine funders of the remaining charities accounting for 96% of this investment. We therefore asked these nine other charities for funding data back to 1970. In many cases, the charities did not have sufficiently robust data management systems to go back that far, and in a number of cases were established at some point during our time series. Furthermore, some had different financial years and different accounting practices (i.e. commitment of multiple year research funding vs. in year expenditure). One charity declined to participate on the grounds that it did not have the resources to collate the information. We worked closely with the other charities to develop our best estimated time series and combined this as ‘other medical research charities’ in our analysis. We deliberately present the aggregate data to protect the confidentiality of the charities and the data they provided. Overall the ‘other medical research charities’ account for approximately 4% of total expenditure on MSD research. For the sensitivity analysis, and to take into account missing data, we increased the other expenditure for the ‘other medical research charities’ by 20% for our high estimate.

### Taking inflation into account

To calculate the total cumulative spend in real terms, the total nominal research spend was adjusted for inflation. We applied a Gross Domestic Product deflator sourced from the HM Treasury (base year = 2013/14) and adjusted total spend for each year based on this [22]. Thus, cumulative funding over the period we examined is expressed in 2013–2014 GBP.

### Royalty payments

In principle, any royalty payments received by research funders as a result of their research investment in the relevant time period should be netted off in the year they occur and so reduce the present value of the investment stream. In previous studies, we had no evidence to suggest that such royalty payments would be sufficient to make a substantive difference to the estimated rate of return. In this case, returns from the royalties relating to the commercial development of anti-tumour necrosis factor (anti-TNF) drugs were believed to be sufficient to have an impact on the IRR. We accessed data from the published annual accounts of the Kennedy Trust and data supplied by ARUK to illustrate, in a sensitivity analysis, the magnitude of the effect of these royalties.

### Estimating the NMB from MSD-related research

This element of the study required estimates of the lifetime quality adjusted life years (QALYs) gained and the net lifetime costs to the NHS of delivering those QALYs for relevant research-based interventions provided in each year of the period 1994–2013. Incremental QALYs encompass both survival and quality of life gains from an intervention as compared to prior practice. We used QALYs gained to quantify health gain rather than changes in DALYs. Although DALYs are used in much of the literature on overall burden of disease itself to characterise population health loss, QALYs are the more appropriate (and much more commonly used measure, particularly in the United Kingdom) to characterise the gain from the use of specific interventions. As far as data permitted, the methods and sources used were chosen to provide directly comparable results to those in the two previous studies on the returns on investment on CVD and cancer research [2, 3].

Overall estimates of the QALYs, and the costs to the NHS of delivering them, were built-up by aggregating estimates for a series of specific interventions. As before, this approach required identification of the key relevant MSD interventions and the number of new patients actually receiving them in the NHS in each year of the relevant period and estimates of the discounted life-time QALY gains and net life-time costs per patient resulting from initiation of the intervention. The aggregated QALYs gained were then valued in monetary terms using, as before, a base-case opportunity cost value of a QALY to the NHS of £25,000 – the midpoint value of the National Institute for Health and Care Excellence’s (NICE) threshold range [23]. From this, the similarly aggregated discounted net lifetime NHS costs of delivering that health benefit were deducted to provide the overall estimate of the NMB. Any specific circumstances where data limitations forced deviation from this approach are noted below.

In the absence of any study that had identified and quantified the research-based MSD interventions that had, during the relevant period, contributed most to the United Kingdom population health gain in this area, or to substantial changes in costs, we reviewed sources that might help build an initial view of likely interventions that might be included. Particularly important in this stage were studies identifying the burden of MSD disease in the United Kingdom [24], relevant NICE Pathways [25], and NICE and National Collaborative Centre (NCC) Guidelines [26–29]. With the assistance of ARUK, we then identified key experts (see Acknowledgements) who, through a workshop (November 2015) and subsequent direct one-to-one interactions, helped produce a list of interventions that, in principle, looked appropriate for inclusion. More detailed review of available data and cost-effectiveness evidence was undertaken to confirm the importance of the listed interventions and to establish whether the necessary estimates of net costs and benefits, and levels of usage, were available. Further input from experts was sought (November 2016 to January 2017) to confirm our assumptions, check for any perceived omissions and to validate the emerging findings, after which some final adjustments were made.

### QALY gains and costs of chosen interventions

We identified appropriate published studies that had estimated the cost-effectiveness of the chosen interventions in the United Kingdom. Wherever possible, we used independent studies produced for NICE or for national clinical guidelines and published in the Health Technology Assessment monograph series, or estimates that had been reviewed and accepted by NICE. In some instances, evidence was taken directly from NICE Technology Appraisals or from National Collaborating Centre Guideline modelling. Where more than one relevant study had been undertaken for NICE, we used the most recent to reflect the developing evidence base. Where no such study for NICE was available we sought the most relevant United Kingdom focussed study from published literature.

### Constructing a time series (1994 to 2013) of usage of MSD interventions

To estimate total NMB for the period, per-patient QALY gains and net costs for each intervention were multiplied by the estimated number of new patients who actually received each intervention in each year. We used the following methods to estimate the time series of usage for the selected interventions.

Data on the number of patients receiving procedural interventions (e.g. hip replacements and surgical length of stay) were gathered from Hospital Episodes Statistics [30] available for years 1999–2013. For pharmacological interventions, prescribing data on total annual spend in the NHS over the period of interest was utilised. Two primary sources of net ingredient cost (NIC) were available – (1) Prescription Cost Analysis (PCA) [31] and (2) Hospital Prescribing England (HPE) [32]. Information on the average cost of a regimen was used, as well as accounting for usage across different diseases and indications, to estimate the number of patients receiving the intervention. Finally, estimates of the average duration of treatment allowed an estimation of the number of patients starting treatment in any of the given years of interest. These data were publically available over different time periods (PCA 1998–2013; HPE 2004–2013). Where an intervention was launched before available data, but within the time period, a linear interpolation was performed with usage assumed to be zero the year before launch. Where an intervention was launched prior to 1994 the last known value was carried back. For years where only PCA data were available, but prescribing also occurred in secondary care, a ratio of the last year of available PCA and HPE data was used to uprate years with PCA NIC only. Estimating the number of patients receiving interventions for low back pain (LBP) was approached differently, using general practice data provided for this study from the Clinical Practice Research Datalink (CPRD) [33].

The component figures of numbers of people receiving treatment interventions were mainly derived from data for England. To produce a United Kingdom estimate (needed because research spend data is for the United Kingdom) figures were adjusted by a factor reflecting England’s proportion of the adult United Kingdom population [34]. All cost estimates were adjusted to 2013–2014 prices using the Hospital and Community Health Services Pay and Prices Index [35].

The value placed on the estimated QALYs gained is a critical parameter in estimating the return on research investment. Given that public spending on health research (whether from taxation or from public donations to medical charities) can be seen as a decision to achieve health benefits through research rather than directly through current healthcare, a value for a QALY (resulting from research investment) should arguably reflect the marginal opportunity cost of generating QALYs in the NHS. In the calculation of NMB in this study, as in the previous two studies, we used as the base-case value an operational opportunity cost value of a QALY in the middle of the ‘threshold range’, as used by NICE in its Technology Appraisals of £25,000 [23]. However, this value can be contested; on the one hand, detailed econometric analysis has estimated that the marginal opportunity cost value in recent years has been significantly lower, at approximately £13,000 [36]. On the other, the value that society places on a QALY as recommended for use in quantifying the impacts of government policies is estimated to be £60,000 [37]. In addition to the base-case, we report values from £13,000 to £60,000.

### Analysis of United Kingdom clinical guidelines to estimate elapsed time and rate of attribution

In the previous studies on CVD [2] and cancer [3] research, the references cited in a sample of clinical guidelines were analysed to inform the estimate of the elapsed time between research spend and net health gain, and the proportion of net health gain that could be attributed to United Kingdom research. In the current study on MSD research, we replicated this approach.

In line with the process for identifying musculoskeletal interventions, guidelines were identified and classified in terms of their relevance for inclusion by comparison to Chapter XIII of the ICD-10 disease classification [19]. Based on this inclusion criterion, a total of 22 national guidelines were identified, spanning a range of practice in the field and issued by ten different bodies (Table 1).

**Table 1:** Summary of United Kingdom guidelines included in analysis of elapsed time and attribution

Provider	Guideline	Year
British Association/College of Occupational Therapists	Hand and wrist orthoses for adults with rheumatologic conditions: practice guideline for occupational therapists	2015
British Association/College of Occupational Therapists	Occupational therapy for adults undergoing total hip replacement: practice guideline for occupational therapists	2012
British Pain Society	Guidelines for pain management programmes for adults	2013
British Pain Society	The assessment of pain in older people	2007
British Society for Rheumatology	British Society for Rheumatology and IASP Musculoskeletal Pain Taskforce Guidelines for the integrated management of musculoskeletal pain symptoms	2008
British Society for Rheumatology	British Society for Rheumatology guidelines on standards of care for persons with rheumatoid arthritis	2005
National Institute for Health and Care Excellence	Osteoarthritis (CG.177)	2014
National Institute for Health and Care Excellence	Osteoporosis (CG.146)	2012
National Institute for Health and Care Excellence	Hip fracture (CG.124)	2011
National Institute for Health and Care Excellence	Rheumatoid arthritis in adults: management (CG.79)	2009
National Institute for Health and Care Excellence	Low back pain in adults (CG.88)	2009
National Osteoporosis Foundation	Clinician’s guide to the prevention and treatment of osteoporosis	2014
National Osteoporosis Guideline Group	Guideline for the diagnosis and management of osteoporosis in postmenopausal women and men from the age of 50 years in the United Kingdom	2014
National Osteoporosis Guideline Group	Osteoporosis: clinical guideline for prevention and treatment: executive summary	2014
National Osteoporosis Society	Vitamin D and bone health: a practical clinical guideline for patient management	2013
Royal College of Nursing	Administering subcutaneous methotrexate for inflammatory arthritis	2013
Royal College of Physicians	Pain: complex regional pain syndrome	2012
Royal College of Physicians	Upper limb disorders: occupational aspects of management 2009	2009
Scottish Intercollegiate Guidelines Network	Management of osteoporosis and the prevention of fragility fractures (CG.142)	2015
Scottish Intercollegiate Guidelines Network	Management of chronic pain (CG.136)	2013
Scottish Intercollegiate Guidelines Network	Management of early rheumatoid arthritis (CG.123)	2011
Scottish Intercollegiate Guidelines Network	Management of hip fracture in older people (CG.111)	2009

We used a bespoke computer programme to extract references from the electronic PDF version of each guideline. In seven cases, the automated reference extraction failed (because papers were not referenced in a recognised format). In these instances, references were extracted manually.

Of the 3640 references cited in the 22 national guidelines, 2746 references (75%) were extracted automatically and 894 (25%) manually. References from non-journal sources (which were unlikely to constitute original research) and duplicates within the same guideline were removed, leaving a total of 3237 references. The average age of the papers cited in a clinical guideline has been termed the ‘knowledge cycle time’ [38], which is the average difference between the publication date of the clinical guideline and the publication date of papers cited in the guideline. The knowledge cycle time was calculated for the 22 identified national guidelines, and used to inform the estimated elapsed time.

To estimate the rate of attribution to the United Kingdom, the 3640 extracted references were provided to the Centre for Science and Technology Studies (CWTS)^5^ to be matched to their bibliometric database (which is derived from the Web of Science). Of the extracted references, CWTS was able to match 2804 (84%); 40 additional references were manually matched, for a total of 2844 (85%). Address data was successfully retrieved from Web of Science for 2762 of these papers. This dataset was used to estimate the degree of attribution to the United Kingdom, based on the addresses of all authors of the included papers. These addresses were used as a proxy for the location in which the research was conducted, and so it was possible to estimate the proportion of the research cited in guidelines that was conducted in the United Kingdom. The non-matched references included non-serial outputs, such as books and websites, journals that are not indexed in the Web of Science, papers whose publication pre-dates a journal’s indexation in Web of Science and incorrect references.

### Calculation of the rate of return

Using the four key sources of data summarised in Fig. 1, we can attribute a proportion of the estimated total annual NMB of the MSD health gain as being due to United Kingdom research, and relate an equal number of years of investment to years of NMB, ‘lagged’ by an estimate of the elapsed time between research and benefit. As in the previous studies, we express this return on investment as an IRR, which is effectively the discount rate that would yield a zero net present value. In this application, the formula for the IRR is:

$$ -\sum \limits_{t=1}^{20}\frac{Res\; In{v}_t}{{\left(1+ IRR\right)}^t}\kern0.36em +\sum \limits_{t=1+ Lag}^{20}\frac{NM{B}_t(Attrib)}{{\left(1+ IRR\right)}^t}=0 $$

Where, *Res Inv* is the United Kingdom research spend on MSD in year *t*, *NMB* is the net monetary benefit in year *t* (monetary value of QALYs gained minus costs of delivery), *Lag* is the estimated average years between research spend and health gain, *Attrib* is the proportion of NMB attributed to United Kingdom research and *IRR* is the internal rate of return.

The IRR is convenient in enabling a comparison to be made between non-competing investments of different sizes with different start dates, as well as providing a direct comparison with our previous work.

Given the nature of the numerous necessary judgements involved, the multiple sources of evidence, the multiple parameters, and the many and various layers of estimates and assumptions, a probabilistic sensitivity analysis was not conducted. Even if it were possible, it would not be informative to express all the uncertainty as ranges for each parameter to reflect stochastic uncertainty in our overall estimate. Instead, we present a range of one-way sensitivity analyses that provide an indication of the uncertainty associated with each of the key aggregate parameters that goes into the calculation, namely the size of the research investment, the average elapsed time between research spend and use of the intervention, the magnitude of the NMB, the proportion of the NMB that can be attributed to United Kingdom research, and the effect of netting-off royalty payments from the investment stream.

## Results

### Public and charitable funding of United Kingdom MSD-related research, 1970–2013

**Fig. 2:**
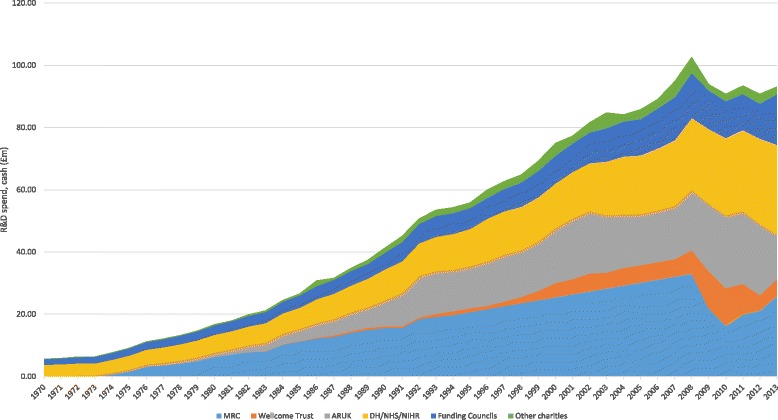
Cash expenditure on MSD research from 1970 to 2013, by funder

**Fig. 3:**
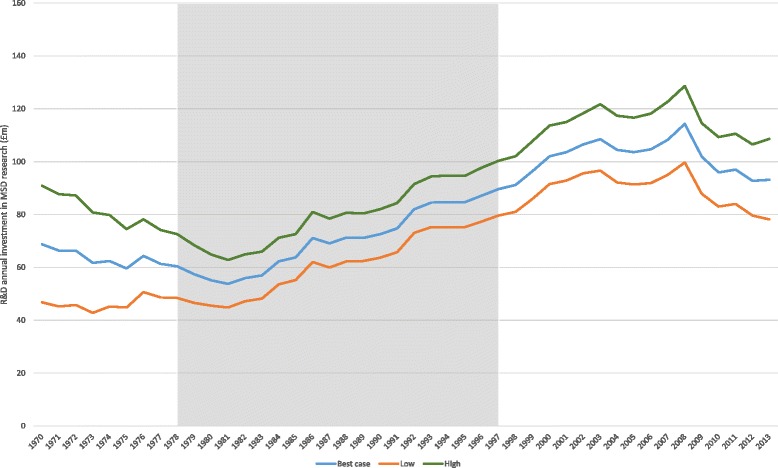
Real term expenditure, 2013 prices, on MSD research 1978 to 1997 (shaded area for 1978 to 1997)

Additional file 1 provides a detailed account of the estimated total expenditure by year by organisation over a 43-year period (1970–2013). A summary of cash expenditure by funder can be found in Fig. 2. The significant spike in 2008 can be attributed to three significantly large grants committed by the WT to the development of large facilities and long-term research programmes. Figure 3 shows the estimated public and charitable expenditure on MSD-related research as £1.4 billion from 1978 to 1997 in constant 2013 prices (i.e. adjusted for inflation). As noted below, 1978–1997 is the funding period used to calculate the IRR taking into account the estimated elapsed time. Figure 3 presents a sensitivity analysis with ‘high’ and ‘low’ scenarios for total MSD-related research spend, with a range of £1.2 billion to £1.6 billion.

We additionally obtained two sets of estimates of royalty payments arising from anti-TNF commercialisation since 2002. The first set was for total royalty payments to the Kennedy Trust and the second was for the sum of the royalties retained by the Trust and those remitted to ARUK. The first is likely to be an over-estimate for our purposes as it includes some royalties received by private individuals; the second may underestimate the total sum that returned into medical research spending. We used both in the sensitivity analyses.

### Interventions

A broad review of the field and discussions with our Advisory Board led us to focus on five main disease areas in which it appeared that the most significant research-based changes to healthcare delivery had occurred between 1994 and 2013. These were inflammatory arthritis, osteoarthritis, connective tissue disorders, osteoporosis and back pain.

**Inflammatory arthritis (M00–M09: RA, JIA and psoriatic arthritis; M10–M12: Gout; M45: Ankylosing spondylitis)**

**Table:** 

**Key interventions:**
• Early, aggressive, combination therapy • Use of anti-TNFs (infliximab, etanercept, adalimumab, golimumab, certolizumab) • Use of other biologics (tocilizumab, abatacept, rituximab) • Allopurinol and febuxostat in treatment of gout

The management of RA and other associated inflammatory arthritis has evolved over the studied time period, predicated by the advent of disease-modifying anti-rheumatic drugs (DMARDs). Conventional DMARDs (cDMARDs), most notably methotrexate, are now a standard component of initial RA management. Treatment has shifted from monotherapy or slow step-up regimen, towards early and aggressive combination therapy (EACT).

Use of methotrexate served as a proxy for all conventional DMARD therapy adjusting for a proportion of methotrexate co-prescribing with biologic DMARDs (bDMARDs). Methotrexate NIC data was only available from the PCA, so the estimates of usage may constitute a slight underestimate. However, given that maintenance doses are normally prescribed in primary care and that patients are likely to receive treatment for 10 years on average [39], most prescribing will be captured in primary care prescribing data. Clinical experts estimated that 40% of patients were managed with EACT in 1994 and essentially all by 2000.

Since the early 2000s the major new treatment option of bDMARDs became available for patients with more severe RA. Anti-TNFs were the initial generation, with other new bDAMRDs emerging that affect RA through other mechanisms.

Estimates for the use of both cDMARD and bDMARD therapy took account of their use in other disease areas (ulcerative colitis, Crohn’s disease, psoriasis, SLE) and across inflammatory arthritis indications (where net health gains are likely to differ). Usage was split proportional to incidence. NICE estimates were used to further allocate bDMARD use across different treatment stages (bDMARD naïve, after failure of an anti-TNF).

Cost effectiveness evidence was available from Tosh et al. [40] for EACT, and was inferred from Stevenson et al. [41] for bDMARDs for RA patients not previously treated with biologics or having failed on cDMARD therapy. Studies by Malkotti et al. [42], Jackson et al. [43] and Minton et al. [44] provided estimates of bDMARD net health gains for patients previously treated with DMARDs including an anti-TNF.

In the absence of indication specific cost-effectiveness data for EACT, its benefits were assumed to be the same for the JIA population and psoriatic arthritis. Shepherd et al. [45] provided estimates of bDMARD net health gains after the failure of cDMARD management and after the failure of an anti-TNF for a JIA population. Rodgers et al. [46] and Corbett et al. [47] were used for bDMARD net health gains in psoriatic arthritis and ankylosing spondylitis, respectively. Beard et al. [48] provided cost-effectiveness data on allopurinol and febuxostat for the treatment of gout.

**Osteoarthritis (M15–M19)**

**Table:** 

**Key interventions:**
• Move to cementless and hybrid hip prostheses • Use of minimally invasive hip and knee replacement • Decreased hospital length of stay for hip and knee replacement from change in surgical management and early rehabilitation • Use of Cox-II inhibitors (celcoxib, etoricoxib, meloxicam, etodolac) • Concomitant Cox-II inhibitor use of proton pump inhibitors (lansoprazole, omeprazole, esomeprazole pantoprazole)

Joint replacement has constituted a mainstay of treatment to alleviate pain and regain function of damaged joints caused by osteoarthritis for some time. As such, it was not appropriate to include all benefits from hip and knee replacement during the period of interest. Incremental changes associated with the use of minimally invasive techniques were, however, relevant to this period, as well as a trend towards cementless and hybrid prostheses for hip replacement. There has also been a marked reduction in hospital length of stay for patients undergoing joint replacement. Mean hospital length of stay for hip and knee replacement surgery in 1999 was 12.7 and 12.3 days, respectively. By 2013, these figures were 5.7 (hip) and 5.1 (knee).

Data from the National Joint Registry [49] was used alongside Hospital Episodes Statistics to estimate the number of minimally invasive hip and knee replacements and type of prostheses used in hip replacement.

Net health gains for cementless and hybrid hip replacements were taken from Pennington et al. [50] and minimally invasive joint replacement from de Verteuil et al. [51]. All hip replacement net health gains were attributed to osteoarthritis, but include a very small proportion of replacement due to other reasons such as RA or dysplasia of the hip. DH reference costs were used to assign a unit cost to a 1-day reduction in length of stay. Savings were estimated by multiplying the annual number of replacements by the difference in length of stay compared to baseline.

All Cox-II inhibitor use was assumed to be in osteoarthritis, with net health gains attributed as such, using evidence from NCC modelling [52]. Cox-II inhibitors taken off the market (i.e. Vioxx) were overall assumed to produce no net benefit. A proportion of concomitant proton pump inhibitor use was assumed over the period, starting at 0% in 2006, rising to 30% by 2012 [52].

**Connective tissue disorders (M30–M35)**

**Table:** 

**Key interventions:**
• Mycophenolate mofetil for SLE

Net health gains resulting from mycophenolate mofetil were available for treatment of SLE [53], although limited to a nephritis population.

**Osteoporosis (M80–M82)**

**Table:** 

**Key interventions:**
• Bisphosphonates (alendronate, etidronate, risedronate, zoledronate) • Hormonal therapies and dual action bone agents (raloxifene, teriparatide, denosmuab, strontium ranelate)

Bisphosphonates, the first of which was launched in the mid-1990s, are recommended as a first-line treatment for post-menopausal osteoporosis and can be used in both primary and secondary fracture prevention. Subsequent hormonal therapies have been developed, which have similar properties.

Some therapies have become generic and these price changes were reflected in the average cost of a regimen over time to estimate the number of patients receiving treatment. However, such changes are not reflected in the cost-effectiveness evidence, and thus will tend to overestimate the lifetime costs of delivering healthcare. Data on zoledronate (Aclasta) NIC was not available in HPE and the manufacturer provided some internal data.

Stevenson et al. [54] provided estimates of per patient net health gains for most of these treatments, although evidence from NICE TA204 2010 [55] was used for zoledronate and denosumab. These data were provided split by age group, and as such net health gains were weighted to reflect the age distribution of the United Kingdom population. Assumptions about the proportion of osteoporosis intervention that is aimed at primary and secondary fracture prevention were taken from NICE estimates [55, 56].

**Back pain (M54)**

**Table:** 

**Key interventions:**
• Manual therapy • Structured exercise programmes • Combined psychological and physical therapy

The focus was primarily on chronic low back pain and sciatica as defined by NICE guidance [57]. In the absence of a comprehensive source through which to ascertain the number of physical and psychological interventions for LBP that patients have received over the period of interest, we had to use a different approach to estimating the population.

Data on new diagnoses of LBP were obtained from the CPRD, which provides observational data from United Kingdom GP practices. Of the Read codes used to identify relevant LBP, approximately 95% of events were one of the following: LBP, back pain without radiation not otherwise specified, sciatica, complaint of LBP, pain in lumbar spine, mechanical low back pain, chronic low back pain, back pain, unspecified, or lumbago. Incidence was defined as the number of incident events divided by the total registered CPRD population (person years) after removing participants who had ever had previous back pain as well as the first year of CPRD sample follow-up, who were defined as not ‘at risk’.

Incidence was split into sex-specific 5-year age bands. United Kingdom population figures were used to estimate a total number of incident cases of back pain during the period based on the CPRD sample. The focus was on a chronic population who receive active interventions over and above self-management and so an assumption around the proportion of incidence that would be chronic in nature was required (40%). Based on NICE guidance [58], Leeds MSK service (personal communication) and a CSAG Report [59] we estimated what proportion of a chronic population would have received each of the three identified interventions over the period of interest. For structured exercise programmes the proportion was estimated to be 5% in 1994 and 20% by 2013; for manual therapy, these figures were 3% and 20%, and for combined physical and psychological therapy 0.3% and 2.5%, respectively.

Data on per patient net health gains were taken from NICE/NCCPC guidelines [58], except for manual therapy, which was taken from a cost-effectiveness analysis of the United Kingdom BEAM trial [60].

### Net monetary benefit (NMB)

Table 2 shows the contribution to the total estimates of lifetime QALYs gained from the nine areas addressed, by year, based on the estimated number of new patients in which the intervention was initiated (procedural interventions are delivered in that year only, but pharmacological treatment duration varies across interventions). By far the largest contribution to the total health gain came from improved treatment of RA (40.1% of the total). Inflammatory arthritis as a whole accounts for 57.5% of the total. Osteoarthritis and osteoporosis are the next biggest areas (21.7% and 12.2%, respectively).

**Table 2:** QALYs gained from key musculoskeletal disease interventions, 1994–2013

	Rheumatoid arthritis (M00–06)	Psoriatic arthritis (M07)	Juvenile idiopathic arthritis (M08–09)	Gout (M10–12)	Osteoarthritis (M15–19)	Connective tissue disorders (M30–35)	Ankylosing spondylitis (M45)	Low back pain (M54.5)	Osteoporosis (M80–82)	Total QALYs
1994	698	67	205	1009	1944	6	0	534	0	4464
1995	1181	113	346	1015	2135	7	0	619	287	5703
1996	1786	171	523	1021	2825	8	0	882	287	7504
1997	2513	241	736	1027	3520	10	0	1171	458	9675
1998	3363	322	986	1033	4219	11	0	1468	572	11,973
1999	4964	499	1270	1038	4736	19	33	1624	853	15,036
2000	6449	641	1705	1089	5249	29	33	1847	915	17,957
2001	8459	845	2288	1128	7010	46	51	2070	1749	23,644
2002	9657	956	2641	1183	9500	63	46	2267	2400	28,713
2003	11,409	1137	3146	1245	11,737	85	69	2499	3663	34,990
2004	14,474	1457	4090	1309	14,454	109	197	2795	5022	43,907
2005	13,976	1454	4053	1157	11,504	106	212	2765	5974	41,201
2006	17,450	1361	3769	1135	11,652	129	154	2747	6833	45,229
2007	19,551	1858	5321	1260	12,929	156	367	2786	7921	52,149
2008	23,730	2381	6309	1493	13,386	181	392	4288	8955	61,116
2009	28,588	2662	7756	1471	13,383	195	482	5741	10,252	70,529
2010	33,882	3071	8945	1592	14,451	219	577	6999	11,994	81,729
2011	45,401	4007	11,994	1797	14,625	169	708	8546	12,538	99,786
2012	51,586	4453	13,422	1885	14,726	125	809	8385	13,111	108,502
2013	50,407	4813	13,248	2143	15,153	128	853	8212	12,875	107,834
Total	349,523	32,507	92,753	26,032	189,136	1801	4983	68,245	106,660	871,693
Value	£8738 m	£813 m	£2,319 m	£651 m	£4728 m	£45 m	£125 m	£1706 m	£2667 m	£21,791 m

Table 3 shows the lifetime net costs to the NHS of new patients initiated on the treatments in question for each of these areas over the 20-year period. Again, by far the biggest costs are associated with RA alone or inflammatory arthritis taken as a whole. It is notable that developments for osteoarthritis have led to a substantial cost saving as a result of lower surgical hospital stay observed over the period. Less substantial, but significant cost savings also arose from treatment of connective tissue disorders, as a result of avoiding the costs associated with renal failure in patients with SLE. The table also reflects that the treatments for LBP that were adopted were relatively cheap and highly cost-effective.

**Table 3:** Net costs of delivery of key musculoskeletal disease interventions, 1994–2013

	Rheumatoid arthritis (M00–06)	Psoriatic arthritis (M07)	Juvenile idiopathic arthritis (M08–09)	Gout (M10–12)	Osteoarthritis (M15–19)	Connective tissue disorders (M30–35)	Ankylosing spondylitis (M45)	Low back pain (M54.5)	Osteoporosis (M80–82)	Total net costs of delivery
1994	£2.0 m	£0.2 m	£0.6 m	£3.3 m	£3.6 m	–£0.3 m	£0.0 m	£5.1 m	£0.0 m	£14.4 m
1995	£3.3 m	£0.3 m	£1.0 m	£3.3 m	£4.2 m	–£0.4 m	£0.0 m	£6.0 m	£4.1 m	£21.9 m
1996	£5.0 m	£0.5 m	£1.5 m	£3.3 m	£10.3 m	–£0.4 m	£0.0 m	£8.5 m	£4.1 m	£32.8 m
1997	£7.0 m	£0.7 m	£2.1 m	£3.3 m	£16.4 m	–£0.5 m	£0.0 m	£11.3 m	£14.0 m	£54.3 m
1998	£9.4 m	£0.9 m	£2.8 m	£3.4 m	£22.5 m	–£0.6 m	£0.0 m	£14.2 m	£16.0 m	£68.4 m
1999	£33.8 m	£3.6 m	£3.6 m	£3.4 m	£26.5 m	–£1.0 m	£1.3 m	£15.7 m	£20.4 m	£107.4 m
2000	£38.0 m	£4.0 m	£4.8 m	£3.6 m	£13.6 m	–£1.4 m	£1.3 m	£17.8 m	£21.3 m	£103.0 m
2001	£53.7 m	£5.5 m	£8.9 m	£3.7 m	£9.1 m	–£2.3 m	£1.7 m	£20.0 m	£36.3 m	£136.6 m
2002	£53.9 m	£5.5 m	£9.1 m	£3.9 m	–£7.7 m	–£3.1 m	£1.6 m	£21.9 m	£44.2 m	£129.3 m
2003	£71.5 m	£7.2 m	£13.7 m	£4.1 m	–£36.0 m	–£4.2 m	£2.1 m	£24.2 m	£64.3 m	£146.8 m
2004	£150.6 m	£13.4 m	£38.0 m	£4.3 m	–£47.7 m	–£5.4 m	£5.1 m	£27.0 m	£87.5 m	£272.8 m
2005	£158.4 m	£15.2 m	£44.9 m	£3.8 m	–£117.8 m	–£5.3 m	£5.5 m	£26.8 m	£108.5 m	£239.9 m
2006	£211.6 m	£11.0 m	£26.2 m	£3.7 m	–£155.2 m	–£6.4 m	£4.5 m	£26.6 m	£122.7 m	£244.7 m
2007	£275.9 m	£21.1 m	£67.8 m	£4.1 m	–£205.5 m	–£7.7 m	£9.3 m	£27.0 m	£140.4 m	£332.4 m
2008	£308.4 m	£27.0 m	£69.4 m	£4.9 m	–£234.4 m	–£9.0 m	£10.2 m	£39.9 m	£157.8 m	£374.3 m
2009	£376.8 m	£27.4 m	£90.4 m	£4.8 m	–£244.1 m	–£9.7 m	£12.5 m	£52.3 m	£176.8 m	£487.3 m
2010	£456.5 m	£31.3 m	£102.9 m	£5.3 m	–£283.0 m	–£10.9 m	£14.9 m	£63.0 m	£202.0 m	£582.0 m
2011	£594.1 m	£37.7 m	£133.5 m	£6.0 m	–£331.7 m	–£8.4 m	£17.9 m	£76.3 m	£211.7 m	£737.2 m
2012	£684.2 m	£41.0 m	£148.2 m	£6.3 m	–£343.8 m	–£6.2 m	£20.3 m	£74.8 m	£220.9 m	£845.6 m
2013	£683.9 m	£53.8 m	£154.3 m	£7.3 m	–£367.6 m	–£6.4 m	£21.6 m	£73.3 m	£215.8 m	£836.0 m
Total	£4178.2 m	£307.3 m	£923.5 m	£85.6 m	–£2268.3 m	–£89.4 m	£129.9 m	£631.5 m	£1868.9 m	£5767.2 m

**Fig. 4:**
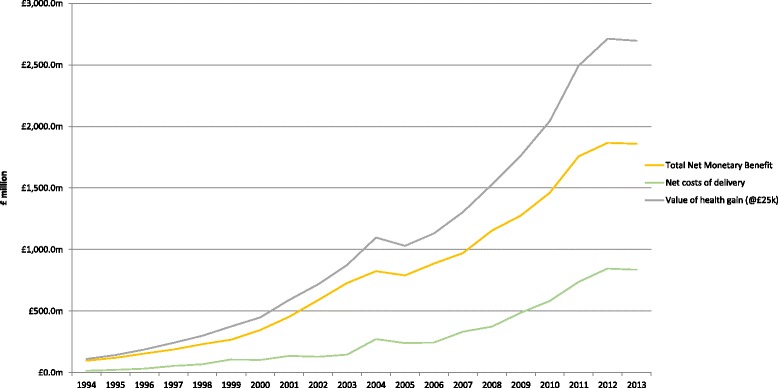
Annual monetised QALYs, net costs of delivery and net monetary benefit – Musculoskeletal disease interventions 1994–2013

Table 4 summarises the NMB when the QALYs have been valued at the base-case value of £25,000 and the net costs to the NHS of the intervention and its long-term sequelae have been deducted. At this value of a QALY, all areas except treatments for ankylosing spondylitis show a positive NMB. Osteoarthritis is the single area with the largest NMB (approximately 43.7% of the total), although inflammatory arthritis as a whole accounts for a similar proportion. Within that total, however, RA accounts for 28.5%. It contributes to NMB to a lesser extent than to QALYs because the new DMARDs have generally been priced to be just acceptable to NICE at the upper end of its £20,000–30,000 ‘threshold’. Indeed, the new DMARDs for RA as a whole produced a negative NMB, but this was offset by large net health gains from the shift towards early, aggressive combination therapy. The overall annual figures for monetised QALYs, net cost of delivery and NMB of key MSD interventions 1994–2013 are shown Fig. 4. Additional file 2 provides details of the breakdown of estimated numbers of patients for each intervention in each year and the related QALY estimates.

**Table 4:** Net monetary benefit from key musculoskeletal disease interventions, 1994–2013

	Rheumatoid arthritis (M00–06)	Psoriatic arthritis (M07)	Juvenile idiopathic arthritis (M08–09)	Gout (M10–12)	Osteoarthritis (M15–19)	Connective tissue disorders (M30–35)	Ankylosing spondylitis (M45)	Low back pain (M54.5)	Osteoporosis (M80–82)	Total net monetary benefit
1994	£15.5 m	£1.5 m	£4.5 m	£21.9 m	£45.0 m	£0.4 m	£0.0 m	£8.2 m	£0.0 m	£97.2 m
1995	£26.2 m	£2.5 m	£7.7 m	£22.1 m	£49.1 m	£0.5 m	£0.0 m	£9.5 m	£3.0 m	£120.7 m
1996	£39.6 m	£3.8 m	£11.6 m	£22.2 m	£60.3 m	£0.6 m	£0.0 m	£13.6 m	£3.0 m	£154.8 m
1997	£55.8 m	£5.3 m	£16.4 m	£22.3 m	£71.6 m	£0.7 m	£0.0 m	£18.0 m	-£2.6 m	£187.6 m
1998	£74.7 m	£7.1 m	£21.9 m	£22.4 m	£83.0 m	£0.8 m	£0.0 m	£22.5 m	-£1.6 m	£230.9 m
1999	£90.3 m	£8.8 m	£28.2 m	£22.6 m	£91.9 m	£1.4 m	–£0.5 m	£24.9 m	£0.9 m	£268.5 m
2000	£123.2 m	£12.0 m	£37.9 m	£23.7 m	£117.6 m	£2.1 m	–£0.5 m	£28.3 m	£1.6 m	£345.9 m
2001	£157.8 m	£15.6 m	£48.3 m	£24.5 m	£166.2 m	£3.4 m	–£0.5 m	£31.7 m	£7.5 m	£454.5 m
2002	£187.5 m	£18.4 m	£56.9 m	£25.7 m	£245.2 m	£4.7 m	–£0.5 m	£34.8 m	£15.8 m	£588.5 m
2003	£213.7 m	£21.3 m	£65.0 m	£27.1 m	£329.4 m	£6.3 m	–£0.4 m	£38.3 m	£27.3 m	£728.0 m
2004	£211.2 m	£23.0 m	£64.3 m	£28.5 m	£409.1 m	£8.1 m	–£0.2 m	£42.8 m	£38.0 m	£824.9 m
2005	£191.0 m	£21.1 m	£56.4 m	£25.1 m	£405.4 m	£7.9 m	–£0.2 m	£42.4 m	£40.9 m	£790.1 m
2006	£224.6 m	£23.1 m	£68.0 m	£24.7 m	£446.5 m	£9.7 m	–£0.6 m	£42.1 m	£48.1 m	£886.1 m
2007	£212.8 m	£25.4 m	£65.2 m	£27.4 m	£528.7 m	£11.6 m	–£0.2 m	£42.7 m	£57.6 m	£971.3 m
2008	£284.8 m	£32.5 m	£88.4 m	£32.5 m	£569.0 m	£13.5 m	–£0.4 m	£67.3 m	£66.1 m	£1153.6 m
2009	£337.9 m	£39.1 m	£103.5 m	£32.0 m	£578.6 m	£14.5 m	–£0.4 m	£91.2 m	£79.5 m	£1276.0 m
2010	£390.5 m	£45.4 m	£120.7 m	£34.5 m	£644.3 m	£16.3 m	–£0.4 m	£112.0 m	£97.8 m	£1461.2 m
2011	£540.9 m	£62.5 m	£166.3 m	£38.9 m	£697.3 m	£12.6 m	–£0.2 m	£137.4 m	£101.8 m	£1757.4 m
2012	£605.4 m	£70.3 m	£187.4 m	£40.8 m	£712.0 m	£9.3 m	£0.0 m	£134.8 m	£106.9 m	£1867.0 m
2013	£576.3 m	£66.5 m	£176.8 m	£46.3 m	£746.4 m	£9.6 m	–£0.2 m	£132.0 m	£106.1 m	£1859.8 m
Total	£4559.9 m	£505.3 m	£1395.4 m	£565.2 m	£6996.8 m	£134.4 m	–£5.3 m	£1074.6 m	£797.6 m	£16,023.8 m

### Estimating the elapsed time

**Fig. 5:**
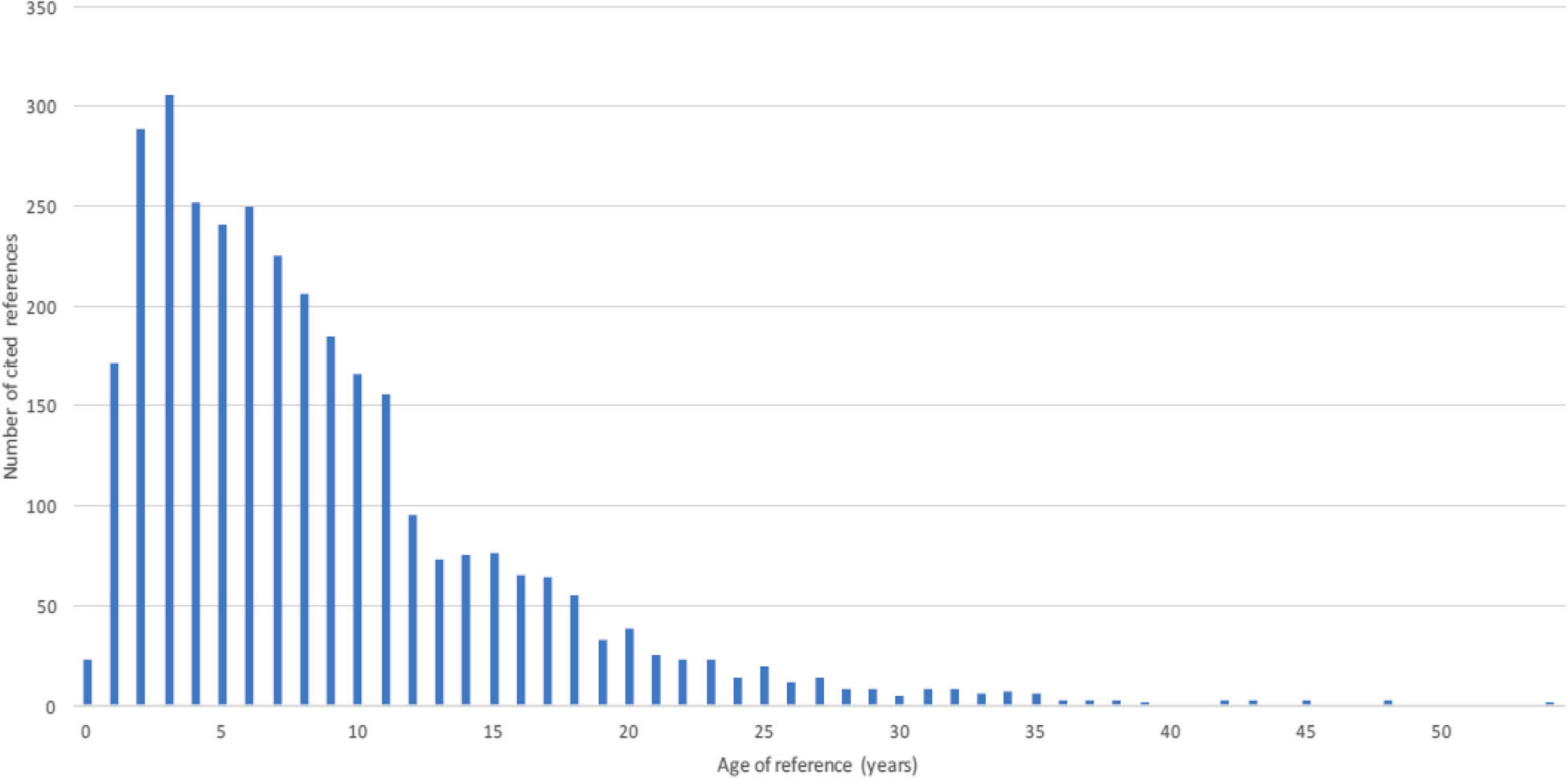
Elapsed time of the cited papers extracted from guidelines

Our estimate of the elapsed time between research funding and health gain was based primarily on analysis of the references cited on clinical guidelines. As illustrated in Fig. 5, the mean age of the 3237 cited papers extracted from the 22 guidelines was 9 years. The median age was 7 years, with an interquartile range of 7 (4–11) years. To produce an estimate of the total elapsed time between investment and return, as required for this study, we added on to this value estimates for (1) the time between the awarding of funding and publication, and (2) the time between recommendation and realisation of health gain in clinical practice. Using the same approach as in our previous studies, we estimated these two periods to total approximately 7 years (3 years for the period between funding and publication and 4 years between recommendation and health gain). This gave a best estimated elapsed time between spending on research and health gain of 16 years. We looked at alternative approaches to estimate the knowledge cycle time, such as only including the NICE and Scottish Intercollegiate Guideline Network guidelines (mean 9 years, median 7 years) and looking at only the main osteoarthritis and RA guidelines, which are the conditions from which the largest health improvements stem (mean 10 years, median 9 years) Additional file 3 provides details of the guideline analysis.

### Estimating the proportion of health gains that can be attributed to United Kingdom research

The estimate of the proportion of the health gain that can be attributed to United Kingdom research was also based primarily on the analysis of cited references on clinical guidelines. A total of 2762 publications were analysed. The overall percentage across all guidelines, using full counting^6^ as for the previous studies, was 30%, which forms our central estimate, but as shown in Table 5, this differed substantially between specific guidelines. We also produced overall estimates using fractional counting^7^ and the reprint address,^8^ which gave an attribution to the United Kingdom of 25% and 24%, respectively.

**Table 5:** Proportion of publications from the United Kingdom for all guidelines included in the analysis

Guideline	United Kingdom papers	Total papers	% United Kingdom
British Association/College of Occupational Therapists – Hand and wrist orthoses for adults with rheumatological conditions: practice guideline for occupational therapists (evidence)	5	25	20%
British Association/College of Occupational Therapists – Hand and wrist orthoses for adults with rheumatological conditions: practice guideline for occupational therapists (supplementary)	10	20	50%
British Association/College of Occupational Therapists – Occupational therapy for adults undergoing total hip replacement: practice guideline for occupational therapists (evidence)	9	30	30%
British Association/College of Occupational Therapists – Occupational therapy for adults undergoing total hip replacement: practice guideline for occupational therapists (supplementary)	1	4	25%
British Pain Society – The assessment of pain in older people	6	63	10%
British Pain Society – Guidelines for pain management programmes for adults	21	55	38%
British Society for Rheumatology – British Society for Rheumatology and IASP Musculoskeletal Pain Taskforce guidelines for the integrated management of musculoskeletal pain symptoms (IMMsPS)	87	304	29%
British Society for Rheumatology – BSR guidelines on standards of care for persons with rheumatoid arthritis	1	1	100%
National Institute for Health and Care Excellence – Hip fracture (CG.124)	73	254	29%
National Institute for Health and Care Excellence – Osteoporosis (CG.146)	32	71	45%
National Institute for Health and Care Excellence – Osteoarthritis (CG.177)	102	416	25%
National Institute for Health and Care Excellence – Rheumatoid arthritis in adults (CG.79)	102	337	30%
National Institute for Health and Care Excellence – Low back pain in adults (CG.88)	31	111	28%
National Osteoporosis Foundation – Clinician’s guide to the prevention and treatment of osteoporosis	33	90	37%
National Osteoporosis Guideline Group – Guideline for the diagnosis and management of osteoporosis in postmenopausal women and men from the age of 50 years in the United Kingdom	2	2	100%
National Osteoporosis Guideline Group – Osteoporosis: clinical guideline for prevention and treatment: executive summary	22	36	61%
National Osteoporosis Society – Vitamin D and bone health: a practical clinical guideline for patient management	25	66	38%
Royal College of Nursing – Administering subcutaneous methotrexate for inflammatory arthritis	14	56	25%
Royal College of Physicians – Pain: complex regional pain syndrome	31	96	32%
Royal College of Physicians – Upper limb disorders: occupational aspects of management 2009	13	52	25%
Scottish Intercollegiate Guidelines Network – Management of hip fracture in older people (CG.111)	41	102	40%
Scottish Intercollegiate Guidelines Network – Management of early rheumatoid arthritis (CG.123)	23	83	28%
Scottish Intercollegiate Guidelines Network – Management of chronic pain (CG.136)	56	171	33%
Scottish Intercollegiate Guidelines Network – Management of osteoporosis and the prevention of fragility fractures (CG.142)	92	317	29%
TOTAL	832	2762	30%

To produce a range of values for the sensitivity analysis, we can consider the potential sources of uncertainty in these estimates. We identify two likely sources of error.

Firstly, we assume, for the purposes of our analysis, that the proportion of research conducted in the United Kingdom corresponds to the proportion supported by United Kingdom (charitable or public) funding. However, United Kingdom authors may receive funding from the United Kingdom or overseas industry or from other non-United Kingdom sources (notably the European Commission, but also other international funders). Equally, United Kingdom funders may fund researchers overseas, but we expect this to be limited in this field, and in most cases this is likely to be in collaboration with at least one United Kingdom author, in which case the full counting model would capture the resulting publications. For the purposes of our model, we assume flows of funding into and out of the United Kingdom to be equal. Considering industry funding, it may be that some of the papers with a United Kingdom address are industry funded (including non-United Kingdom industry), and as such should be excluded from the number of United Kingdom papers for our estimate of attribution to the United Kingdom. We expect this proportion to be small, but this is clearly an issue which warrants further investigation.

Secondly, there is uncertainty around the relative contribution of funding associated with each author (and hence country) listed on each paper. The three bibliometric methods all estimate this differently. Using full counting effectively assumes that the United Kingdom contributes all the funding for any paper which has a United Kingdom author (and does the same for any other countries on the same paper). This is likely to overestimate the United Kingdom contribution. Fractional counting at the author level assumes an equal contribution of funding from each author on a paper (from the country in which they are based). With reprint addresses, the assumption is that all funding comes from the country in which the corresponding author is based. For the last two approaches, it is not clear whether they are likely to give an under- or overestimation of the proportion of funding from the United Kingdom. For consistency with previous studies, we have used the full counting approach for our central estimate.

Based on this analysis, we conclude that it is unlikely that the United Kingdom contribution is higher than our estimate from full counting of 30% (as used in previous studies). However, it may be lower than our lowest estimate using the reprint addresses of 24%, considering the other potential sources of funding available to United Kingdom-based researchers. Taking this into account, we used lower and upper bounds of 20% and 30% for the sensitivity analysis.

### Estimating the IRR from musculoskeletal disease research

Our estimates of the NMB produced by year (summarised in Table 4) at a base-case value of a QALY of £25,000 were then related to our best estimates of public and charitable spend by year on MSD research (summarised in Fig. 3) and expressed as an IRR. Calculation of the IRR incorporates our best estimates of the average elapsed time between research spending and use of the intervention (16 years) and of the proportion of the NMB that could be attributable to United Kingdom research (30%). This gives a base-case estimate of an IRR of 6.8%.

As is evident from the methods used, there is inevitably considerable uncertainty around the values of all our estimates. Table 6 presents a series of one-way sensitivity analyses to illustrate the effects of some of the main areas of uncertainty – all changes have predictable effects. Despite the detail of our estimation process there is considerable uncertainty in the NMB; we present the implications for the IRR of an arbitrary but plausible range of −25% and +25% around our estimate to reflect this. We also present the IRR omitting the cost-savings from reduction in length of stay for hip and knee replacements (see Discussion). The impact of taking into account the royalty payments arising from anti-TNF research increased the IRR by 0.2 percentage points taking our lower figures (possible underestimate) and by 0.4 percentage points using our higher figures (likely overestimate).

**Table 6:** Internal rate of return: one way sensitivity analyses

	IRR
Best Estimate	6.8%
Low research spend (£12 m)	7.6%
High research spend (£16 m)	6.0%
Omit length of stay reduction	5.5%
QALY £13 k	0.8%
QALY £20 k	5.0%
QALY £30 k	8.1%
QALY £60 k	12.9%
Long lag (20 years)	5.5%
Short lag (11 years)	8.1%
Low attribution to United Kingdom (20%)	4.5%
NMB −25%	5.1%
NMB +25%	8.0%
Royalty payments to public/charitable funders	7.0%
Total royalty payments	7.2%

*IRR* internal rate of return, *QALY* quality adjusted life years, *NMB* net monetary benefit

The IRR predictably decreases with increased estimates of research funding and elapsed time and, as far as is explored, all the variables in the one-way sensitivity analyses show a positive rate of return. However, in combination, they could of course have produced a wider range of estimates for the IRR. Table 6 shows that, inevitably, the IRR is most sensitive to the range of plausible values that can be placed on the value of a QALY. At an opportunity cost in the NHS of £13,000, the IRR falls to 0.80%, whilst at a societal valuation of £60,000 the IRR is 12.9%.

## Discussion

In this paper, we have estimated the economic returns from public and charitable funding of MSD-related research in the United Kingdom. Expressed in 2013 prices, total expenditure on MSD-related research was £1.4 billion for the period (1978–1997) that was used to estimate the rate of return. Over the period 1994–2013, the key interventions we analysed produced 871,000 QALYs with a NMB of £16 billion, allowing for the net NHS costs resulting from them and valuing a QALY at £25,000. The proportion of benefit attributable to United Kingdom research was 30% and the elapsed time between funding and impact of MSD treatments was 16 years. Our best estimate of the IRR from MSD-related research was 7%, very similar to the 9% for CVD research and 10% for cancer research (Table 7). When combined with previous estimates of the broader economic (or ‘spillover’) benefits of biomedical and health research in the United Kingdom of 17% [4], the total rate of return is approximately 24–27%.

**Table 7:** Comparison of key results with previous studies

	MSD	Cancer	CVD
Average annual research investment (for years of data used in IRR calculation as reported in source publications, using different time period for calculating constant prices and therefore not suitable for comparisons)	£70 m (1978–1997, in constant 2013–2014 prices)	£266 m (1976–1995, in constant 2011–2012 prices)	£111 m (1975–1998, in constant 2005–2006 prices)
Average annual research investment (rebased in same constant prices for comparative purposes)	£70 m (1978–1997, in constant 2013–2014 prices)	£290 m (1976–1995, in constant 2013–2014 prices)	£133 m (1975–1998, in constant 2013–2014 prices)
Elapsed time (between spending on research and health gain)	16 years	15 years	17 years
Attribution (proportion of papers that include a United Kingdom address from the papers cited on guidelines)	30%	17%	17%
Average NMB (for years of data used in IRR calculation as reported in source publications, but using different time period for calculating constant prices therefore not suitable for comparisons)	£801 m (1994–2013, in constant 2013–2014 prices)	£6223 m (1991–2010, in constant 2011–2012 prices)	£2949 m (1992–2005, in constant 2005–2006 prices)
Average NMB (rebased in same constant prices for comparative purposes)	£801 m (1994–2013, in constant 2013–2014 prices)	£6458 m (1991–2010, in constant 2013–2014 prices)	£3559 m (1992–2005, in constant 2013–2014 prices)
IRR (health gain)	7%	10%	9%

*CVD* cardiovascular disease, *IRR* internal rate of return, *MSD* musculoskeletal disease, *NMB* net monetary benefit

In this study, we have also further tested the bottom-up methodological approach developed in the original ‘Medical Research: What’s it worth?’ study [2]. The application of this method to a further disease area that is different to CVD and cancer – particularly in terms of the chronic nature of MSD and the predominantly quality of life gains of the benefit of the interventions – confirms the generalisability of our approach to estimate the economic returns from research; that is not to say that it is without limitations. We have organised the discussion on limitations and caveats by first looking at the key conceptual issues with the methodological approach, followed by issues related to data availability and quality, then an examination of a set of issues related to MSD research, and, finally, a set of key caveats on what this work demonstrates and what it does not.

### Key conceptual assumptions inherent to methodological approach

In estimating the economic returns from MSD-related research (and indeed in the previous studies looking at CVD and cancer research) various key assumptions are made that are intrinsic to the conceptual approach adopted:

- **The total NMB for the interventions not covered is assumed to be zero.** Our estimate assumes that any other MSD interventions introduced or widely adopted during the period in question not included in the analysis have, in aggregate, no effect on the NMB, that is, their NMB is equivalent to zero. Put another way we assume that, for any omitted interventions, the monetised value of the health benefit is equal to the cost of delivering the benefit. This seems a reasonable assumption as there may be interventions where the cost of delivery outweighs the value of the benefit and others where the value of the benefit outweighs the costs of delivery. Without analysing all these other interventions, it would be wrong to speculate on the balance of these effects and therefore they are assumed to cancel each other out. However, as discussed below, the likelihood of this assumption being correct will vary as the value of the QALY is decreased or increased in the sensitivity analysis.
- **The total net flow of knowledge between disciplines is assumed to be zero.** We know that the relationship between research discipline and impact is ‘many-to-many’ [61], that is research from a specific discipline will contribute to multiple types of impact and a specific impact is often made up of contributions from multiple research disciplines. In the context of the current study, it is likely, for example, that MSD-related research benefits from, say, cancer research and vice versa. We therefore assume that the flow of knowledge is the same in to as it is out of different research fields, in effect cancelling each other out.
- **The definition of MSD-related research used by the research funders captures basic research.** We know this is the case for ARUK and the other disease-specific medical research charities as all their research funding is included in the analysis. For the FCs and the DH/NHS this would not be an issue as estimates for their MSD-related research funding were derived by applying the ‘activity index’, which would include basic research. However, for the MRC and WT this could be an issue. For the MRC, we relied on the funder’s classification and used the broader of two definitions so that we would deliberately err on the side of caution by taking the higher level of R&D spend. For the WT, we had to rely on search terms and in scanning research grant titles we were reassured that fundamental research was included.
- **The cost of private sector R&D is covered in the net NHS costs of the interventions.** We assume that the costs of private sector R&D (i.e. non-public and non-charitable research expenditure) are accounted for when we net off the NHS costs for an intervention. This assumption holds for purely commercial research as, say, a pharmaceutical company will include the cost of their R&D investments in the price of a drug. It may be that companies invest in ‘non-commercial’ activities, such as public–private partnerships or precompetitive consortia, and in effect are subsidising the public sector research in doing so. However, even in this case (which is probably at the margins of total R&D investments) it is unlikely that the private sector is doing so in isolation of commercial considerations and it will recoup such costs through its sales revenues.
- **All health gains arise from specific patient interventions.** We assume that all health gains arise from, and are captured in, our estimates of the health gain from specific patient interventions. We recognise that broader service changes, such as the adoption of fracture clinics, or improvements in diagnosis are important but assume that they lead to patients receiving timely and appropriate interventions for which we estimate the QALYs gained. There is a possibility that we are failing to net off the full costs of such developments in service delivery if the cost-effectiveness evidence we use for such interventions fails to reflect the full cost of the service delivery associated with them.
- **We have assumed there is a causal relationship between research and health gains.** Our analysis relies on the assumption that the health benefits would not have occurred without the evidence from the medical research. Our bottom-up approach has the advantage that, for the individual interventions, there is causality as demonstrated through their formal clinical trials. Additionally, in this disease area we do not have the uncertainty as to any causal factors, other than medical research, that may have led in part to a reduction in smoking. Furthermore, in previous research on MSD, we used a case study approach that clearly demonstrated causality for the small number of interventions examined [62, 63].

### Uncertainties relating to key parameters

There remain a number of uncertainties with the bottom-up approach that relate to the nature, quality and availability of data that are relevant to this examination of MSD research, and were also the case for CVD and cancer research. Reducing the uncertainty with these data issues would improve the robustness of the IRR estimate and thus, in part, could inform future research avenues.

- **The monetary value of a QALY.** As noted earlier in the paper, there is ongoing debate as to the appropriate value of a QALY. Our base-case assumption of £25,000 is consistent with our analysis of the returns to CVD and cancer research, and reflects the mid-point in the range of values (of £20,000 to £30,000) cited as normal criteria for acceptance of interventions by NICE [24]. However, as highlighted above, if the QALY is valued either at a lower (e.g. £13,000) or higher (e.g. £60,000) level then this could affect our core assumption that the total NMB for any new interventions not covered is assumed to be zero. If QALYs are valued at £60,000 then more interventions are likely to have a positive NMB among those not looked at, meaning that we are underestimating the rate of return. Conversely, if the QALY is valued at £13,000, then more interventions in those not looked at are likely to have a negative NMB, meaning that we are over estimating the rate of return.
- **Estimates of the elapsed time are hard to determine.** As with the previous studies, bibliometric analysis of clinical guidelines was used to estimate the time between research investment and health gain. The advantage of this approach is that it provides empirical estimates, but it is also, inevitably, a gross simplification of a complex and varied process, as we have discussed elsewhere [64, 65]. The estimate of the elapsed time is in accordance with other estimates using different approaches as reviewed by Morris et al. [66], but is still a crude proxy and is an area that would benefit from further research.
- **Estimates for the rate of attribution are very hard to determine.** Like the estimate of the elapsed time, bibliometric analysis of clinical guidelines was used to estimate the proportion of the United Kingdom health gains that can be related to United Kingdom public and charitable research funding. However, the estimate of the attribution rate is harder to validate than the elapsed time and is thus more contestable. It is also becoming increasingly difficult to define, given the steady increase in international collaboration in research observed in recent decades [67]. Identifying any one country’s contribution without a qualitative assessment of the research itself can only provide an uncertain estimate, but one that we believe is likely to be more robust at the aggregate level of an entire research field. Biases in coverage of bibliometric databases, particularly as regards languages other than English, should also be noted. An attribution rate of at least 7–9% would be expected given that the United Kingdom contributes approximately 7–9% of biomedical and health research outputs [68]. One could also argue that the rate would be somewhat higher than this given that the local healthcare context is likely to drive the need for locally relevant studies. In the previous two studies, the attribution rate was 17% for both CVD and cancer (Table 7), which, given the above logic, felt defensible. However, an attribution rate of 30% for MSD seems high and was at the top end of the estimates we generated using different bibliometric methods. Thus, and although we used 30% for consistency with the previous studies, we did not include a higher upper bound estimate in the sensitivity analysis. It may also be that a proportion of papers cited on the clinical guidelines are solely private sector-funded and thus overstate the attribution rate to publically funded research (a scan of the references suggests that approximately 10% of United Kingdom papers could be solely industry funded). Either way a clear priority for future research would be to further examine how you measure how much of the United Kingdom health gain you can attribute to United Kingdom public and charitable research, and to validate or otherwise the guideline methodology.
- **Missing funding data.** Historical data on research funding expenditure was incomplete, meaning that we had to make a number of assumptions to account for missing data. These assumptions erred on the side of caution and were tested in the sensitivity analysis of the IRR. As we have previously noted [2], if research funders wish to carry on with this type of analysis, the continued use of standard systems of research classification such as the HRCS will be important.

### Key issues particular to MSD

There are a number of specific issues that relate to the assessment of MSD research, as indicated below.

- **Quality of data.** We had expected that there might be greater problems in identifying cost-effectiveness data in MSD given that the outcomes of interventions are principally improvements in quality of life. In practice, there was relatively good data on the cost-effectiveness and usage of new drugs, reflecting that many had been subject to NICE appraisals. By contrast, there was much less cost-effectiveness data and very poor data on provision and usage for some interventions such as those for back pain. In part, this reflects the complexity and variability of the physical therapies potentially provided to multiple groups of MSD patients. There are issues about the generalisability of clinical trials and associated cost-effectiveness studies and an absence of consistent routine methods of data collection on their usage.
- **Pricing of new pharmacological interventions.** One of the major therapeutic developments in the period was the advent of biologic DMARDs including anti-TNFs. Our analysis shows that these made a substantial contribution to the QALYs gained, but as most were priced to try to meet NICE’s cost-effectiveness ‘threshold’ they contributed rather little to our estimates of NMB. However, as biosimilars now become available these drugs will, in future, if not superseded by other new interventions, contribute more strongly to any estimate of NMB. By contrast, early aggressive treatment with generic methotrexate provides both QALYs and a high NMB.
- **Importance of reductions in length of stay.** Our clinical experts emphasised the importance of changes in practice during the relevant period that had reduced lengths of stay and hence costs for some key procedures. They noted that, whilst desire for the reduction may have been driven by cost considerations, the change in practice was supported by research evidence showing no reduction in health benefit [69–71]. Review of the data showed that the change was marked and we estimated the cost savings in the case of hip and knee replacements. However, we are aware that there may have been some similarly marked changes that we did not quantify in previous studies. Whilst we do not believe they would have been so significant in the case of CVD or cancer we provide a sensitivity analysis to show the IRR for MSD if these cost savings are excluded.
- **Other interventions that we might have included.** Some advances in the treatment of musculoskeletal connective tissue disorders have occurred over the period of interest, but data on their cost-effectiveness is limited. For example, there was a lack of cost-effectiveness data for other immunosuppressant therapies in SLE and scleroderma, notably cyclophosphamide and intravenous immunoglobulin for the treatment of dermatomyositis. The net health gains associated with rituximab use in SLE were also not quantified in the model due to a lack of data. Similarly, we were unable to characterise the cost-effectiveness and to find appropriate data on specific treatments for soft-tissue musculoskeletal pain (M60–M79). As noted above, for any area we were not able to analyse specifically, our methods implicitly assume that any benefits from treatment were directly offset by their costs of delivery (i.e. the NMB is 0). However, we are confident that our analysis directly captures most of the significant advances in the field that have produced important health or cost effects when viewed at a population level, and unlike our analysis of cancer, because the MSD field is smaller, we did not have to prioritise and effectively ignore some potentially important areas.
- **The treatment of royalty payments.** The particular circumstances of the commercialisation of anti-TNF research led us to consider for the first time in this study the impact on the IRR of the significant royalty payments, although we were unable to establish the precise total magnitude of the royalty payments that were returned to publicly funded medical research. The impact was not negligible although they did not significantly change the order of magnitude of the IRR. We are not aware of royalties of a similar relative magnitude in the case of our previous studies on CVD and cancer, but clearly it is an issue that needs to be considered and refined in future studies.

### Key limitations and caveats to the ‘bottom-up’ approach for assessing economic returns

There are three key caveats that are fundamental to appropriately using the results presented in this and the previous papers in assessing the economic rates of return for MSD, cancer and CVD research.

- **We have assessed past performance, not predicted future performance.** In all three studies, we have estimated the rate of return based on past investments and therefore our results cannot be a guarantee of future returns – medical research does not advance in a smooth and linear fashion. This is a crucial caveat when using these results to advocate the need for future research spending.
- **We have assessed the average rate of return, not the marginal rate of return.** From the analysis, we are not able to say whether the rate of return would have been different if research spending had been higher or lower, or whether at the margin the returns to research investment are increasing or decreasing. Assessing the marginal rate of return is of clear interest to policy-makers and this is a topic that warrants further research attention.
- **Our estimates should not be used to make comparisons between disease area or intervention.** Given the inherent assumptions and uncertainty in our approach we strongly counsel against making comparisons between the three disease areas we have examined or specific interventions within those disease areas. Taking the three studies together, we believe it is appropriate to say the IRR arising from health gains to the United Kingdom, from United Kingdom research, is between 7% and 10%.

It is of course impossible to know whether our estimates of the return are accurate in the absence of an observation of a control to provide the counterfactual. The likely validity of our estimates can only be judged through the reasonableness of the many assumptions about which we have tried to be entirely transparent. Should readers have more or less confidence in them because of the similarity between the estimates for IRR arising from health gains across the three studies? The results are indeed remarkably comparable (Table 7). This can either be interpreted positively as some form of internal validation of plausible magnitudes, or negatively to suggest that something inherent in the methodology leads to this similar outcome. We are not aware of any aspect of our methods that suggests this is a methodological artefact. Rather, we draw some comfort from the fact that the similarity arises despite the inputs (research expenditure) and outcomes (NMB) being very different between the studies and for separate groups of interventions within each of them. The fact that the elapsed times are similar has a degree of face validity, as does the relatively high level of attribution for MSD, which, as already noted, given its chronic nature, is more likely to be influenced by local contextual research.

## Conclusion

The public, both as taxpayers and charity donors, invest a significant amount of money into biomedical and health research each year. Understanding the economic impact of this investment provides accountability, helps secure future research investments and increases our understanding of how research is effectively translated into health improvements. In a series of studies looking at the net value of improved health outcomes, in CVD, cancer and MSD we have demonstrated an IRR of between 7% and 10%. When we include the 17% return for the broader economic or ‘spillover’ impact this rises to between 24% and 27%. The results suggest that, despite the uncertainties around the methods and estimates, the historical returns in terms of NMB of the health gains derived in the United Kingdom from public and charitably funded biomedical and health research are substantial and justify the investments made.

## Additional files

Additional file 1: Appendix 1. Funding data. (XLSX 21 kb)

Additional file 2: Appendix 2. Health gain (i.e. net monetary benefit) data. (XLSX 61 kb)

Additional file 3: Appendix 3. Guideline data. (XLSX 16 kb)

## Abbreviations

AMRC: Association of Medical Research Charities
Anti-TNF: anti-tumour necrosis factor drugs
ARUK: Arthritis Research UK
bDMARDs: biologic disease-modifying anti rheumatic drugs
cDMARDs: conventional disease-modifying anti rheumatic drugs
CPRD: Clinical Practice Research Datalink
CVD: cardiovascular disease
CWTS: Centre for Science and Technology Studies
DALYs: disability adjusted life years
DH: Department of Health
DMARDs: disease-modifying anti rheumatic drugs
EACT: early and aggressive combination therapy
FC: Funding Councils
GP: general practitioner
HPE: Hospital Prescribing England
HRCS: Health Research Classification System
ICD: International Statistical Classification of Diseases and Related Health Problems
IRR: internal rate of return
JIA: juvenile idiopathic arthritis
LBP: low back pain
MRC: Medical Research Council
MSD: musculoskeletal disease
NCC: National Collaborative Centre
NHS: National Health Service
NIC: net ingredient cost
NICE: National Institute for Health and Care Excellence
NMB: net monetary benefits
PCA: prescription cost analysis
QALY: quality adjusted life years
RA: rheumatoid arthritis
R&D: research and developments
SLE: systemic lupus erythematosus
WT: Wellcome Trust.

## Footnotes

1 The Health Research Classification System (HRCS) is a two-dimensional framework. Codes from both HRCS dimensions are applied when classifying; one dimension, the Health Categories, is used to classify the type of health or disease being studied. There are 21 categories encompassing all diseases, conditions and areas of health. The other dimension, the Research Activity Codes, classifies the type of research activity being undertaken (from basic to applied). There are 48 codes divided into eight groups. See http://www.hrcsonline.net/rac/rac for more details. The data cited are on DALY rates from 2012 and research spend data from 2014.

2 Figures given between 1992 and 1993 show the UFC grants after the funds were transferred from the UFC to the Research Councils.

3 Prepared by the HEFCE Analytical Services Group on 13 February 2008. Figures for 1993–1994 to 1996–1997 were adjusted to include an estimate of funds for research capital that were rolled into mainstream quality-related research (QR) from 1997–1998 onwards. No adjustments have been made to counterbalance the effect of the phased transfer of funds to the United Kingdom Research Councils in 1992–1993, 1993–1994 and 1994–1995. Figures for Units of Assessment (UoA) 12: Biochemistry, which ceased to exist in the 2001 Research Assessment Exercise, have been rolled into UoA 14: Biological Sciences. Figures exclude funds for the supervision of students on research degree programmes, London weighting, and all other relatively minor elements of research funding. For 2006–2007, 2007–2008 and 2008–2009, the QR charity support element has been added to mainstream QR funds to reflect the change in the way research income from charities is used in the calculation of funding. Figures contain relatively minor grant adjustments made to the database after the initial grant allocations announced in March each year.

4 Figures for 2010–2011 do not reflect the 1.7% retrospective reduction announced in HEFCE Circular Letter 05/11 and applied at institution level. In the calculations that include quality-related research (QR) charity support funding (below), the proportion of QR charities support funding attributed to biomedical Units of Assessment in 2011–2012 has been applied to 2012–2013.

5 The Centre for Science and Technology Studies (CWTS) maintains a bibliometric database of all scientific publications (including health and biomedical research) for the period 1981 to 2016. This dataset is based on the journals and serials processed for the Core Collection version of the Web of Science database, including the Science Citation Index Expanded (SCI-E) and associated citation indices, the Social Sciences Citation Index (SSCI), and the Arts & Humanities Citation Index (A&HCI). This database is operated for bibliometric purposes in service contracts under a License Agreement with Clarivate Analytics. See: https://www.cwts.nl/ for more information.

6 Full counting is an approach in which a paper which has authors from multiple countries will be attributed in full to each of these countries. Therefore, the estimate of 30% indicates that the United Kingdom contributed to 30% of the papers analysed. However, there will be contributors from other countries to many of these papers. This approach is used for the central estimate as it was used in the previous studies, to aid comparison.

7 In fractional counting, attribution of a paper is shared between the countries of origin of the various authors. For example, if a paper has one author from the United Kingdom and two from the United States, the United Kingdom will receive attribution for one third of a paper, and the other two thirds will go to the United States.

8 This is an alternative approach where papers are attributed to the country of origin of the corresponding author. The logic is that it is likely that the institution of the corresponding author held a significant proportion of the funding for the work and made a significant contribution to the work.
